# The Contribution of Vegetation and Landscape Configuration for Predicting Environmental Change Impacts on Iberian Birds

**DOI:** 10.1371/journal.pone.0029373

**Published:** 2011-12-22

**Authors:** Maria Triviño, Wilfried Thuiller, Mar Cabeza, Thomas Hickler, Miguel B. Araújo

**Affiliations:** 1 Department of Biodiversity and Evolutionary Biology, National Museum of Natural Sciences, CSIC, Madrid, Spain; 2 Laboratoire d'Ecologie Alpine, UMR CNRS 5553, Université Joseph Fourier, Grenoble, France; 3 Metapopulation Research Group, Department of Biological and Environmental Sciences, University of Helsinki, Helsinki, Finland; 4 Department of Physical Geography and Ecosystems Analysis, Geobiosphere Science Centre, Lund University, Lund, Sweden; 5 Biodiversity and Climate Research Centre (BiK-F) and Department of Physical Geography at Goethe-University and Senckenberg Gesellschaft für Naturforschung, Frankfurt/Main, Germany; 6 ‘Rui Nabeiro’ Biodiversity Chair, CIBIO, University of Évora, Évora, Portugal; 7 Center for Macroecology, Evolution and Climate, University of Copenhagen, Copenhagen, Denmark; Smithsonian's National Zoological Park, United States of America

## Abstract

Although climate is known to be one of the key factors determining animal species distributions amongst others, projections of global change impacts on their distributions often rely on bioclimatic envelope models. Vegetation structure and landscape configuration are also key determinants of distributions, but they are rarely considered in such assessments. We explore the consequences of using simulated vegetation structure and composition as well as its associated landscape configuration in models projecting global change effects on Iberian bird species distributions. Both present-day and future distributions were modelled for 168 bird species using two ensemble forecasting methods: Random Forests (RF) and Boosted Regression Trees (BRT). For each species, several models were created, differing in the predictor variables used (climate, vegetation, and landscape configuration). Discrimination ability of each model in the present-day was then tested with four commonly used evaluation methods (AUC, TSS, specificity and sensitivity). The different sets of predictor variables yielded similar spatial patterns for well-modelled species, but the future projections diverged for poorly-modelled species. Models using all predictor variables were not significantly better than models fitted with climate variables alone for ca. 50% of the cases. Moreover, models fitted with climate data were always better than models fitted with landscape configuration variables, and vegetation variables were found to correlate with bird species distributions in 26–40% of the cases with BRT, and in 1–18% of the cases with RF. We conclude that improvements from including vegetation and its landscape configuration variables in comparison with climate only variables might not always be as great as expected for future projections of Iberian bird species.

## Introduction

Global environmental changes pose great challenges to biodiversity, with ongoing impacts on species distributions and abundances already being recorded (e.g. [1]–[3]). Attempts to estimate the future effects of global change on biodiversity have often relied on environmental envelope models [4]. These models relate known species distributions to environmental variables to project future altered potential distributions under global change scenarios (e.g. [5]–[7]). Most of the studies have used climatic factors alone to project species distributions into the future. Nevertheless, there are many factors other than climate that can affect the geographical distributions of species (e.g. [8], [9]). This is particularly true for animal species for which climate is often used as a surrogate for resource availability or nesting suitability.

A large number of studies have included non-climatic factors for modelling contemporary species distributions. Such factors included, among others, land cover and land use [10]–[12], vegetation cover [13], topography [14], or a combination of all of them [15]. However, only a small number of assessments exploring the potential impacts of future global environmental changes have included predicted land use or vegetation changes to complement climatic information (but see [16]–[19]) because of the scarcity of relevant non-climatic data projected into the future. To our knowledge, none of these previous studies has incorporated vegetation dynamics modelled in a mechanistic way as we have done in this study. The question remains: how would changes in non-climatic environmental factors affect projections of future altered species distributions? We address this question using Iberian birds as a case study.

European bird species have already shown phenological (e.g. [20], [21]) and distributional changes (e.g. [22], [23]) and they are projected to shift their ranges substantially as a result of global change [24]. However, improvements of projections of future range shifts could be expected if information on vegetation dynamics was included because bird species distributions are known to be, at least partially, determined by vegetation and its spatial configuration (e.g. [25]–[27]). Variables characterizing aspects of vegetation have been used to model potential current distributions of birds (e.g. [13], [28]), but they have rarely been incorporated in models projecting future range shifts under scenarios of global environmental change [29]. Furthermore, most attempts to incorporate vegetation dynamics into forecasts of species distributional changes have not considered vegetation dynamics, such as those simulated by Dynamic Vegetation Models (DVMs), but rather used statistical interpolation of vegetation patterns [18], [30]. For example, Lawler et al [29] simulated changes in the vegetation distribution with the Mapped Atmospheric-Plant-Soil System (MAPSS), an equilibrium model that provides future static snapshots, but no year-to-year variability. The spatial configuration of vegetation cover is also thought to be important for explaining bird distributions (e.g. [31], [32]), because it accounts for the amount of available habitat in the surrounding area, but again little attempts have been made to incorporate landscape dynamics in forecasts of biodiversity change.

In this study, we used distribution data for 168 breeding bird species in the Iberian Peninsula to fit models using combinations of climatic variables, vegetation characteristics, and their derived landscape configuration. Models were used to assess the importance of alternative aspects of the environment for projecting future potential bird ranges. Specifically, we address the following questions: (i) what sets of variables have greater predictive power: climate, vegetation or landscape configuration? (ii) Are projections using different environmental predictor variables coincident?

## Materials and Methods

### Species data

We used distributional records in the Iberian Peninsula for 168 native breeding bird species. Distribution data were extracted from the Spanish Atlas of Breeding Birds [33] and from the Portuguese Atlas of Nesting Birds [34] reporting the presence and absence of bird species in 5923 10×10 km resolution UTM cells. This is the highest-resolution animal distribution data available for the Iberian Peninsula. Our analyses of bird distributions excluded marine and aquatic species because modelling of their habitats would require information about variables that is not available to us. Species with less than 20 records were also excluded to avoid problems of modelling species with small sample sizes [35].

### Environmental data for the baseline period

Variables were selected from a larger pool based on expert knowledge and data mining; the latter was done with the specific goal of reducing the number of variables and remove collinearity among them. Overall, four groups of continuous predictor variables were used to fit the models (Table 1): (i) *climatic* (3 variables), (ii) *vegetation* (17 variables), (iii) *landscape configuration* (3 variables) and (iv) *global* (including all previous variables).

**Table 1:** Environmental variables used to build alternative models.

Variable name	Variable description
***Climate data set***	
1 mwintertmp	Mean winter temperature (°C)
2 annpre	Annual precipitation (mm)
3 acmgddaug	Accumulated degree days (January to August)
***Vegetation data set***	
*Forest type*	
1 Bet.pen	*Betula pendula*
*2* Bet.pub	*Betula pubescens*
3 Car.bet	*Carpinus betulus*
4 Cor.ave	*Corylus avellana*
5 Fag.syl	*Fagus sylvatica*
6 Fra.exc	*Fraxinus excelsior*
7 Pic.abi	*Picea abies*
8 Pin.hal	*Pinus halepensis*
9 Que.ile	*Quercus ilex*
10 Que.pub	*Quercus pubescens*
11 Que.rob	*Quercus robur*
12 Til.cor	*Tilia cordata*
13 Total.Forest	Sum of all the forest types
*Shrubland type*	
14 MRS	Mediterranean Raingreen Shrub4
15 Jun.oxy	*Juniperus oxycedrus*
16 Que.coc	*Quercus coccifera*
*Grassland type*	
17 c3	Herbaceous
***Landscape data set***	
1 Forest.R30	Accumulated forest in a radius of 30 km
2 Shrub.R30	Accumulated shrubland in a radius of 30 km
3 Grass.R30	Accumulated grassland in a radius of 30 km

For the (i) ***climatic group***, a set of aggregated climate parameters were derived from the Climate Research Unit at 10′ resolution. The CRU CL 2 and CRU CL 2.1 dataset at resolution of 10′ (∼16 km at the latitude of the study) was chosen to represent current climate (average from 1971 to 1990). Average monthly temperature and precipitation in grid cells covering the mapped area of the Iberian Peninsula were used to calculate mean values of three different climate parameters: mean winter temperature, annual precipitation and accumulated degree days. These variables are considered ecologically important for explaining bird distribution patterns (e.g. [36]–[38]) and limit species distribution as a result of widely shared physiological constraints (e.g. [39], [40]). Finally, variables were interpolated using kriging implemented within Geographical Information System (GIS) software ArcGIS 9.2 [41] to a resolution of 10 km to match the bird distribution datasets.

The (ii) ***vegetation group*** comprised potential natural vegetation composition and structure, simulated with the DVM LPJ-GUESS [42], [43]. The model simulates the competition between main tree species and PFTs. Forest dynamics resemble successional patterns, adopting a forest “gap model” approach. The model has been parameterized to represent the main European tree species and a number of plant functional types (PFTs) [44], [45]. LPJ-GUESS reproduced the main general patterns in European potential vegetation at a coarse scale, but the model did not reproduce the fine-scale mosaic of different vegetation types existing in many areas. Discrepancies were, for example, caused by the fact that some real-world drivers, such as different soil nutrient levels, are not accounted for by the model. However, the model results we used present the first assessment of dynamic future vegetation changes at the level of important tree species and PFTs over continental Spain and Portugal. General vegetation features in the Iberian Peninsula, such as the distinction between forests, shrublands and grasslands, corresponded better with the potential natural vegetation in the Iberian Peninsula than in earlier studies with dynamic global vegetation models. [45]. The model also reproduced the main features of the coarse-scale distribution of major tree species covered by the Third Spanish Forest Inventory [46] (Figure S1). The PFTs were also grouped into three broad habitat types, reflecting the vegetation structure rather than individual tree species or PFTs: forest, shrubland, and grassland. The sum of the LAI of all species and PFTs belonging to each of the three broad habitat type group was then used in the analyses. Many bird species are rather dependent on such structural vegetation features than on individual tree species [25], [26], [47]. Furthermore, the model output for these structural ecosystem features is more robust than the simulated patterns for individual species or PFTs, and they are less likely to be fundamentally changed by forest management. A PCA was performed in order to investigate for collinearity among variables and potentially select a reduced set of variables. However, variables were not highly correlated so all were kept. The vegetation was represented by the continuous variable Leaf Area Index (LAI), which is the ratio of total upper projected leaf surface of vegetation divided by the surface area of the land on which the vegetation grows. LAI is a dimensionless value, typically ranging from 0 to 8 for a dense forest. The variables were originally at 10′ (∼16 km at the latitude of the study) resolution and were interpolated at 10 km resolution to match the bird distribution datasets.

Because potential vegetation cover variables modelled with LPJ-GUESS do not account for current and future land use, we combined them with land use information derived from CORINE Land Cover (CLC) as follows [48]. Categories from CLC were aggregated and represented by 6 land cover classes: *Urban*, *Cropland*, *Permanent Crops*, *Grasslands*, *Forest* and *Others* (for a complete description of the methodology see [49], despite in this reference they use the PELCOM dataset, the analyses were re-done using CORINE dataset and are the ones used for this study). The percentage of each land use type within the UTM grid cells was calculated using the Zonal Statistics tool implemented in ArcGIS 9.2. Grid cells were classified as forested when 10% or more of their surface were covered by *Forest*. If, for example, the vegetation model predicted forest but less than 10% of the grid cell was forested according to the land cover data, non-forest vegetation cover was assumed in the analysis. From the grid cells classified as shrublands we excluded the ones in which the sum of non-compatible land use types (*Permanent Croplands*, *Croplands* and *Urban*) represented 90% or more of the grid area. Finally, cells were classified as grasslands when their area was covered by at least 10% of *Grasslands*. Thus, we assume that a certain fraction of available habitat within a grid cell is sufficient for populations to persist. Different classes were not exclusive between each other and grid cells could hold more than one vegetation type at the same time. If, for example, a grid cell was covered by 17% of forest and 16% of grassland according to land cover data and was occupied by *Quercus ilex* (PFT of forest type) and c3 (PFT of grassland type) according to the vegetation model, that grid cell was considered both as “forest” and as “grassland”.

The (iii) ***landscape configuration group*** was calculated based on the accumulated sum of the different PFTs values included in each habitat type: forest, shrubland, and grassland. Using ArcGIS 9.2., three concentric bands, each 10 km wide, were delimited around each grid cell for the three habitat types. Within each band and for each habitat type, the accumulated vegetation abundance was calculated. These data provided information of the spatial arrangement and composition of the landscape around each grid cell. From the nine variables created only the three variables of radius equal to 30 km were retained due to the high correlation between the three different radiuses (Spearman's correlations, *r* = 0.8–0.9) and also because they capture a broader range of landscape and were the variables least correlated with the original habitat types.

Finally, the (iv) ***global group*** included the three previous data sets.

### Environmental data for the future

We used a European climate scenario from the EU framework program Assessing Large-scale environmental Risks for biodiversity with tested Methods (ALARM) at a resolution of 10′ for the period 2051–2080 [50]. The climate scenario was derived from a simulation with the global climate model HadCM3, using the BAMBU (Business As Might Be Usual) scenario (which corresponds to A2 SRES) of the ALARM project. Scenarios for future potential natural vegetation were developed by a previous study [45] as well as the scenarios for future land use change [51]. Land use projections used to constrain potential vegetation cover from LPJ-GUESS were based on the BAMBU scenario [52] (for details see [51], [53]).

### Data analysis

The models were built using the BIOMOD library [54] in R [55] (version 1.15), using the default settings and parameters. Two ensemble modelling techniques were selected: Random Forests (RF) [56], [57] and Boosted Regression Trees (BRT) [58], [59]. Both techniques are effective in dealing with non-linearities and interactions among variables. Random forest uses a bootstrap aggregation algorithm by fitting multiple un-pruned classification trees on sub-samples of the original data. The prediction is then the average of the predictions of all trees weighted by their internal predictive accuracy (out-of-bag estimator). We fitted random forest using a maximum of 700 trees and using a random half of the predictor variables for each tree. BRT is a boosting algorithm in which very short classification trees (seven nodes) are repeatedly built on the residuals from the previous tree to improve the fit using cross-validation to stop the process. In BRT models the maximum number of trees was set to 3000, the learning-rate was 0.001 and the interaction-depth was 4 as suggested by Elith et al. [58]. The full dataset for the 168 breeding bird species was randomly partitioned into two subsets (calibration and evaluation), with 70% and 30% respectively, and this overall procedure was repeated five times to make sure that the evaluation procedure was independent of the random splitting procedure. Future projections were made assuming unlimited dispersal, which is a more likely scenario among birds at the geographical extent of the study area than the alternative no dispersal scenario.

**Figure 1:**
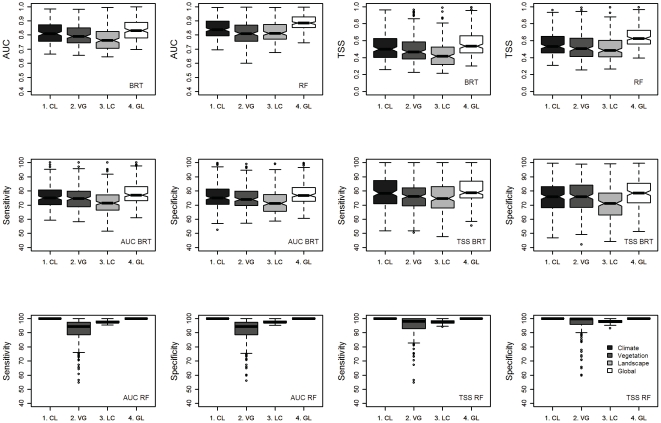
Four evaluation methods to compare model performance using different predictor variables. Boxplot summarizing results of measures of performance (AUC and TSS) of each dataset used (Climate, Vegetation, Landscape and Global) for the cross validation results for BRT and RF models. Percentage of presence and absence correctly predicted (sensitivity and specificity) were also provided. Median values (line across box), range excluding outliers (error bars), interquartile range containing 50% of values (box) and outliers (circles) from results. Untransformed values have been used.

Models were assessed using four evaluation methods: the area under curve (AUC) of the receiver operating characteristic (ROC) [60], the true skill statistics (TSS) [61], sensitivity that measures the percentage of presences correctly predicted and specificity that measure the percentage of absences correctly predicted. The specificity and sensitivity were determined separately after using an AUC and TSS protocol to convert probabilities of occurrence into presences and absences (Figure 1).

There is a large number of statistical techniques available to fit environmental envelope models and they are known to produce markedly different future projections of species range shifts when projections are made into the future [62]–[64]. Commonly used evaluation metrics measuring agreement between predicted potential and observed distributions are useful to verify the models' discrimination ability [63]. However, discrimination between predicted potential and observed distributions is known to be a relatively poor surrogate of the models' ability to predict future distributions well [65]. Therefore, there are little guidelines for selection of the models to use under future scenarios [66]. A possible approach to handle inter-model variability and reduce uncertainty is to use ensemble forecasting by generating multiple copies of the models and combining them using consensus techniques (see for review [66]). In this study, a consensus approach based on the mean of the probabilities from the sets of projections made by RF and BRT was selected (see also [67]–[69]) and TSS method was chosen to convert probabilities values into presence-absence data.

The relative importance of environmental variables was also calculated for RF and BRT. In Random Forests, variable importance is determined by comparing the misclassification error rate of a tree with the error rate that occurs if the values of a predictor variable are randomly permuted [57]. In Boosted Regression Trees variable importance is based on the number of times a variable is selected for binary splitting, weighted by the squared improvement to the model as a result of each split, and averaged over all the individual trees [70]. Because measures of variable importance are calculated differently in RF (Mean Decrease Accuracy and Mean Decrease Gini) and BRT, a ranking system was created to compare environmental selection among the different model types. Environmental variables were ranked from 1 (most important) to 23, although only the three first ones were analysed to compare across all groups of variables (only three variables for the climatic group).

Bird species were classified into eight categories based on their main habitat use: Forest, Shrubland, Grassland, Grassland/Forest, Shrubland/Forest, Grassland/Shrubland, Grassland/Shrubland/Forest and Others (including bird's species which do not depend on any vegetation type such as those specialized on urban areas or cliffs). In order to define the degree of habitat specialization of species we counted the number of habitat types used for breeding or feeding and considered that the more habitats used the less specialized are the species. The information was gathered from the Spanish Atlas of Breeding Birds [33] and complemented by consultation with experts (Table S1).

## Results

Average discrimination ability of models based on cross validated AUC and TSS values differed statistically among the different groups of predictor variables (Friedman test, *p*<0.001), being lower for landscape models and higher for models including all predictor variables together (Figure 1). Models including climatic variables alone were generally better than models fitted solely with vegetation or landscape variables, although not always significantly better than models including vegetation (Wilcoxon test, *p*<0.05) (Table 2). The comparison between the models including all variables and the models including climate, vegetation or landscape showed that the all-variables models were significantly better than any other model, except for the models fitted with climatic variables alone for which the all-variables-model was significantly better only in 50% of the cases (Table 3). Regarding the differences in discrimination ability between modelling techniques, we found that Random Forests adjusted projections to the data more closely than Boosted Regression Trees in almost all of the cases and regardless of the four evaluation techniques used (Figure 1).

**Table 2:** Results of pairwise Wilcoxon test of the effect of predictor variables (climate, vegetation and landscape configuration) on model performance estimated by AUC and TSS.

Predictor variables	Climate-Vegetation				Climate-Landscape				Vegetation-Landscape			
Evaluation method	AUC		TSS		AUC		TSS		AUC		TSS	
Model technique	BRT	RF **	BRT	RF	BRT ***	RF **	BRT ***	RF **	BRT **	RF	BRT **	RF
Wilcoxon test	*W* = 13512	*W* = 14459	*W* = 13326	*W* = 13410	*W* = 15853	*W* = 14168	*W* = 15727	*W* = 14155	*W* = 14528	*W* = 11319	*W* = 14595	*W* = 12470
	*p* = 0.0575	***p*** ** = 0.0019**	*p* = 0.096	*p* = 0.077	***p*** ** = 1.1e-06**	***p*** ** = 0.0063**	***p*** ** = 2.5e-06**	***p*** ** = 0.0066**	***p*** ** = 0.0014**	*p* = 0.3801	***p*** ** = 0.001**	*p* = 0.562

**Table 3:** Results of pairwise Wilcoxon test comparison between each individual model (climate, vegetation and landscape configuration) and the global model based on performance estimated by AUC and TSS.

Predictor variables	Climate-Global				Vegetation-Global				Landscape-Global			
Evaluation method	AUC		TSS		AUC		TSS		AUC		TSS	
Model technique	BRT	RF ***	BRT	RF***	BRT ***	RF ***	BRT ***	RF ***	BRT ***	RF ***	BRT **	RF ***
Wilcoxon test	*W* = 9839	*W* = 7566	*W* = 10030	*W* = 7666	*W* = 8258	*W* = 5940	*W* = 860	*W* = 6878	*W* = 6396	*W* = 5737	*W* = 6568	*W* = 5889
	*p* = 0.0059	***p*** ** = 1.8e-08**	*p* = 0.01203	***p*** ** = 3.6e-08**	***p*** ** = 2.0e-06**	***p*** ** = 1.4e-14**	***p*** ** = 1.6e-05**	***p*** ** = 7.7e-11**	***p*** ** = 1.1e-12**	***p*** ** = 1.8e-15**	***p*** ** = 5.2e-12**	***p*** ** = 8.5e-15**

**Figure 2:**
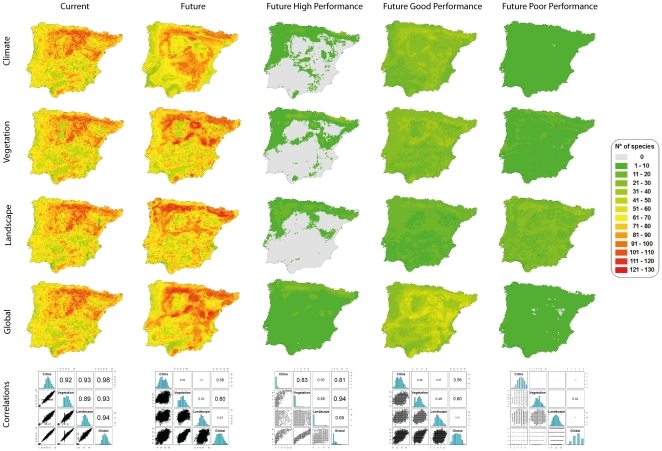
Spatial pattern comparison of bird distributions. The maps represent the total number of species per each 10 km cell for the four model types (Climate, Vegetation, Landscape and Global) and for two time periods (current and future projections). The correlation graphs indicate the level of agreement between the four model types for each column. The calculations for the first two columns (current and future) were done using the total number of bird species (N = 168) whereas the last three columns illustrate subsets of the future projection based on model performance categories (AUC method): high (N = 32), good (N = 63) and poor (N = 37).

Spatial correspondence among projections of species richness for the four sets of models was very high for the baseline period, but substantially variable for future scenarios. Inter-model variability was constrained by model performance (Figure 2). That is, species for which models performed notably well (*high-performance species*) had lower inter-model variability than species for which models performed well (*good-performance species*) and poorly (*poor-performance species*) (Table 4). Overall, the pairwise correlation among future projections for the 168 species varies considerably (Spearman's correlations, *r* = 0.26–0.8). However, pairwise comparisons for groups of species with models of similar accuracy (grouped according to AUC values) showed that higher correlation between model predictions was obtained for the models with higher accuracy: *high-performance species* (Spearman's correlations, *r* = 0.5–0.94; maximum number of species = 32); *good-performance species* (*r* = 0.37– 0.6; maximum number of species = 63); and *poor-performance species* (*r* = 0.17–0.44; maximum number of species = 37).

**Table 4:** Number of species from the 168 species classified in different accuracy classes of AUC and TSS based on two modelling techniques.

Predictor variables	Climate				Vegetation				Landscape			
Evaluation method	AUC		TSS		AUC		TSS		AUC		TSS	
Model technique	BRT	RF	BRT	RF	BRT	RF	BRT	RF	BRT	RF	BRT	RF
High-performance	28	35	17	19	23	27	15	21	17	28	5	10
Good-performance	61	78	28	32	53	60	21	25	35	71	19	30
Fair-performance	70	53	79	90	78	46	80	74	75	59	66	85
Poor-performance	9	2	44	27	14	28	51	48	41	5	78	43
Fail	0	0	0	0	0	7	1	0	0	5	0	0

**AUC:** High = AUC>0.9, Good = 0.9<AUC<0.8; Fair = 0.7<AUC<0.8; Poor = 0.6<AUC<0.7. Fail AUC<0.6.

**TSS:** High = TSS>0.8, Good = 0.8<TSS<0.6; Fair = 0.6<TSS<0.4 and Poor = 0.2<TSS<0.4. Fail TSS<0.4.

**Figure 3:**
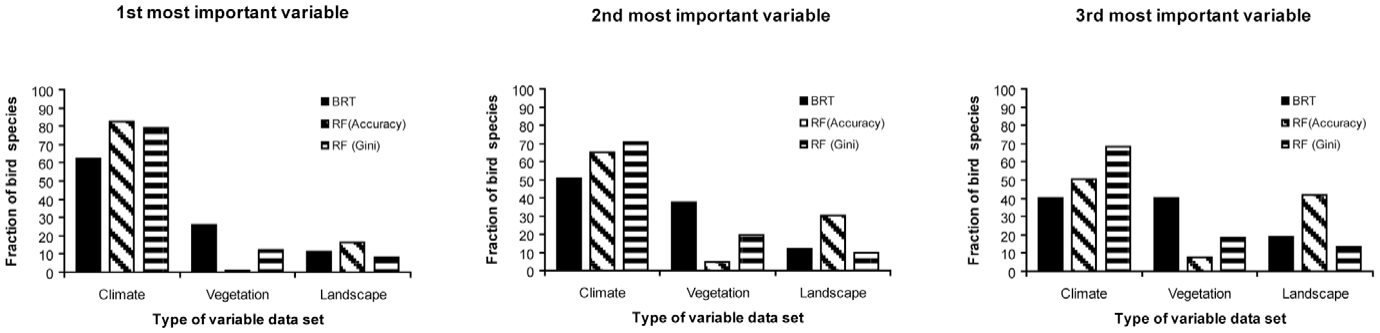
Ranking of variable importance for BRT and RF models. Fraction of the 168 bird species for which the model selected climatic, vegetation or landscape variables as the first, the second or the third most important variable.

After ranking the relative importance of all the environmental variables, we calculated the fraction of species for which the models selected climatic, vegetation or landscape variables among the three most important ones. Results were different depending on the method used (Figure 3). Using the procedure for assessment of variable importance in BRT, we found that vegetation was selected as important for a larger fraction of bird species (26.2–40.5%) than that estimated with RF models (Accuracy 1.2–7.7%, and Gini index 12.5–18.4%). For the three measures of variable importance used (BRT, Accuracy and Gini index), the fraction of species for which the models selected non-climatic variables increased from the first most important variable (1.2–26.2%) to the second (4.8–37.5%) and third variable selected (7.7–40.5%).

**Figure 4:**
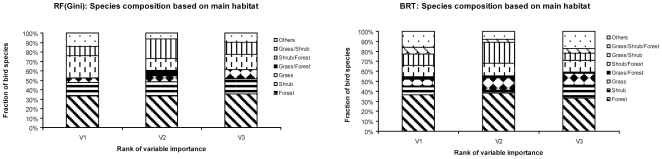
Importance of vegetation variables among bird species with different habitat preferences. Species composition based on the main habitat used by the bird species selecting vegetation variables as the first, second or third most important for explaining their distribution. For BRT model species number for V1 = 44, V2 = 63 and V3 = 69 whereas for RF model (Mean Decrease Gini measure) the species number for V1 = 21, V2 = 33 and V3 = 31.

The main type of habitat used by the bird species was not associated with the choice of variables entering into the models (Figure 4) neither did the degree of habitat specialization (Table 5). As it can be seen in figure 4, vegetation variables were selected as the first, second, or third most important variable for a constant fraction of bird species. For example, vegetation was associated with ∼35% of forest bird specialists in all cases. Unlike the expectation, no clear variable discrimination emerged in models using vegetation variables among forest, shrubland or grassland birds.

**Table 5:** Fraction of bird species for which the model included vegetation variables as the first (V1), second (V2) or third (V3) most important variables.

Model technique	BRT			RF (Gini index)		
Specialization level	V1	V2	V3	V1	V2	V3
**High specialization**	52.3%	54%	58%	52.4%	54.5%	61.3%
**Mid specialization**	25%	34.9%	20.3%	33.3%	39.4%	29%
**Low specialization**	6.8%	3.2%	4.3%	0%	0%	0%

Species are grouped by their degree of habitat specialization based on the number of habitat types they use for breeding and feeding. **High specialization** means the species use one habitat type (N = 90), **mid specialization** means the species use two habitat types (N = 51) and **low specialization** means the species use three habitat types (N = 9).

## Discussion

In this study we asked whether adding vegetation and landscape configuration variables in environmental envelope models would significantly increase discrimination ability of models and whether different sets of variables would affect the spatial representation of climate change impacts on bird species. We showed that models using climatic variables generally fit the data better than models using vegetation or landscape configuration variables. However, improvements of discrimination with the climate models, as compared with the two alternative models, were significant in all cases only for the climatic-landscape model comparison. Disagreement existed between future projections using different predictors, but the discrepancy decreased when species with high levels of discrimination ability in ensembles of forecasts were retained. Finally, the importance of variables appeared to be species specific and, despite the importance of climatic variables, vegetation and landscape configuration were also important for explaining the distribution patterns of a number of bird species.

### Climatic variables perform better than non-climatic variables when predicting potential distributions of birds

Authors have repeatedly suggested that greater care should be given to the choice of environmental predictors when modelling the potential distributions of species (e.g. [71]). Previous studies have suggested that non-climatic variables should be incorporated in bioclimatic models for projecting future range shifts (e.g. [13], [72]), but the impossibility of validating future projections [65], [73] makes it complicated to measure the relative importance of non-climatic variables. It is well-established that the configuration and composition of vegetation are good predictors of bird species distributions because they are associated with many of their breeding, feeding or nesting requirements (e.g. [74] and references therein). For example, Seoane et al. [13] found that vegetation models were significantly more accurate than topo-climatic models. However, our results showed that vegetation or landscape models did not outperform climatic models. Indeed, for half of the modelled species consideration of all variables did not result in better discrimination than that obtained with models only accounting for climate variation. Possible explanations for this result are that: (i) the relative importance of climatic versus non-climatic predictors is scale dependent (e.g. [75]). For example, in a previous study, land cover data did not improve model accuracy at coarse resolution (50 km) in Europe [11]. In another study, using a finer resolution (10 and 1 km), the inclusion of land use improved model discrimination ability [12]. In effect, the resolution and extent of our study might be too coarse to capture the dependence of birds on vegetation; (ii) vegetation in Mediterranean countries has been modified by humans for millennia. The human impact is not represented by the simulated potential vegetation. We sought to address this issue by tailing vegetation to land use, but the land cover data used herein is still a rather coarse approximation of real land cover and its associated habitat characteristics. However, the correspondence between species potential distributions and simulated potential vegetation might be higher in regions where the actual vegetation has been little influenced by human activities; (iii) the vegetation model used here was parameterized to represent the main dominant tree species and vegetation types across Europe, but it did not include all important trees in the Iberian Peninsula. Furthermore, as with any process-based vegetation model, simulated vegetation patterns do not always correspond well with real patterns; (iv) the coarse vegetation and land use variables used in this study do not account for all important habitat characteristics, such as forest age and size structure in plantations and the amount of deadwood.

Discrepancies between future projections could be partly explained by the expected decrease in the correlation between climate and simulated vegetation across time. This is because, firstly, the vegetation model accounts for potential effects of increasing atmospheric CO_2_ on productivity and water cycling [44], [76]. “CO_2_ fertilization” and reductions in stomatal conductance and water losses might alleviate some of the negative effects of increasing drought on vegetation [44], [77]. Secondly, the vegetation model simulates transient vegetation shifts, not the equilibrium response to the climatic forcing. Over a few decades, only a small fraction of the long-term equilibrium response of the vegetation can be expected [45]. This non-equilibrium is much more important for the discrepancies in the projections than the CO_2_ effects [44], [45].

### Species characteristics influence model accuracy

Species characteristics have been shown to influence model accuracy and many biological traits such as body size or dispersion rate and also population trends have been measured for evaluating their influence on modelling results [78]. Species with narrower or spatially more aggregated ranges (e.g. [79], [80]) and higher habitat specialization (e.g. [81], [82]) can generally be predicted with higher accuracy. Our results support the conclusions from these studies, as the species with the highest accuracy values across all model types (climate, vegetation, landscape configuration and global) included high-mountain species with very narrow ranges and low prevalence, such as Tengmalm's owl *Aegolius funereus*, bearded vulture *Gypaetus barbatus*, rock ptarmigan *Lagopus mutus*, capercaillie *Tetrao urogallus* and ring ouzel *Turdus torquatus*. In our study, the ranking of species by accuracy values was similar across models as it was shown when future projections for the subgroup of species with good model performance were compared (Figure 2). Therefore, other relevant environmental or biological predictors might be required for those species that were difficult to model.

### The importance of predictors is species specific

It is difficult to determine what are the most important environmental variables constraining species distributions, especially when a large number of species is considered. Nevertheless, we note that most of the divergence in future projections was caused by species that were difficult to model with our predictors, i.e., that performed poorly with the measures of discrimination ability used to verify model performance. Models discriminating data well yielded less variable projections into the future. More work is needed to identify whether animal species can be grouped based on their response to global environmental changes as well as identify which functional traits made them more resistant to these changes.

We conclude that the discrimination ability of envelope models is not always improved by inclusion of vegetation and landscape configuration variables. In the particular case of bird species in the Iberian Peninsula, climate was sufficient to describe current distributions for ca. 50% of the species and in some of the remaining cases vegetation could help improving the fit of the models but not landscape configuration. With our data and analysis, no general patterns emerged with regards to the selection of vegetation variables by models of different guilds of species. So, the decision as to whether to include specific non-climatic factors in the models requires case specific considerations based on the auto-ecology of the species.

## Supporting Information

Figure S1 **(A) Comparison between the simulated LAI of the first five main tree species (** ***Betula pendula*** **,** ***Corylus avellana*** **,** ***Fagus sylvatica*** **,** ***Fraxinus excelsior*** **and** ***Quercus robur*** **) and presence data from the Third Spanish Forestry Inventory (IFN = Inventario Forestal Nacional).** Inventory data was not available for all simulated tree species. The first column of maps represents the model outputs, the second column the result from the combination of LPJ-GUESS results with a land use dataset (see Materials and Methods for further details), and the third column represents the presence data of the IFN. The model reproduced the broad distinction between northern and southern trees, but the simulated distribution of more northerly distributed species generally expanded further to the south than according to the inventory data. This was too some extent expected as the model represented potential natural vegetation. The Mediterranean region has a long history of large-scale anthropogenic impacts. Most areas once occupied by forest were transformed into croplands and pastures hundreds and in many cases even thousands of years ago (e.g. [83]), while the rest of the remaining forest has been intensively managed [84]. Also the imposition of real land use patterns could only partly remove this mismatch because the land use data only distinguished forest and non-forest areas, without tree species-specific information. As a result, the simulated distribution was maintained in the simulated data as long as the land use data indicated that the forest cover was, at least, 10% (see Materials and Methods). Another explanation for the wider simulated ranges might be that the inventory might not cover all small outlier populations. (**B**) Comparison between the simulated LAI of the last five main tree species (*Picea abies*, *Pinus halepensis*, *Quercus ilex*, *Quercus pubescens* and *Tilia cordata*) and presence data from the Third Spanish Forestry Inventory (IFN = Inventario Forestal Nacional).
(TIF)

Table S1 **Main habitats (G = grassland, S = shrubland, F = forest, O = others) for the 168 bird species included in the study.** The information was gathered from the Spanish Atlas of Breeding Birds [33] and complemented by consultation with the following experts: Carlos Ponce, Sergio Pérez Gil and Alejandro Aparicio Valenciano.
(DOCX)
