# Supplementary figures and images for: The Contribution of Vegetation and Landscape Configuration for Predicting Environmental Change Impacts on Iberian Birds
Source: PLoS One. 2011 Dec 22;6(12):e29373. doi: 10.1371/journal.pone.0029373 (PMC3245269; doi:10.1371/journal.pone.0029373)

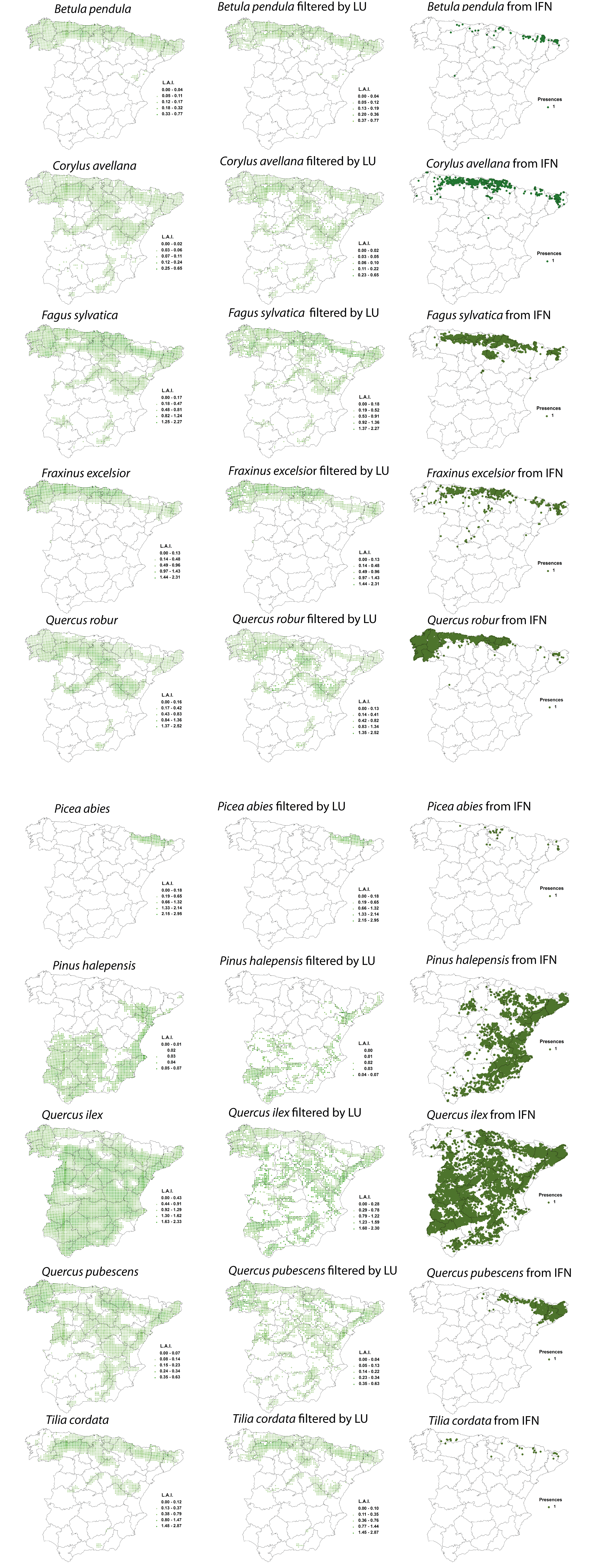

Supplement: Figure S1 — (A) Comparison between the simulated LAI of the first five main tree species ( Betula pendula , Corylus avellana , Fagus sylvatica , Fraxinus excelsior and Quercus robur ) and presence data from the Third Spanish Forestry Inventory (IFN = Inventario Forestal Nacional). Inventory data was not available for all simulated tree species. The first column of maps represents the model outputs, the second column the result from the combination of LPJ-GUESS results with a land use dataset (see Materials and Methods for further details), and the third column represents the presence data of the IFN. The model reproduced the broad distinction between northern and southern trees, but the simulated distribution of more northerly distributed species generally expanded further to the south than according to the inventory data. This was too some extent expected as the model represented potential natural vegetation. The Mediterranean region has a long history of large-scale anthropogenic impacts. Most areas once occupied by forest were transformed into croplands and pastures hundreds and in many cases even thousands of years ago (e.g. [83]), while the rest of the remaining forest has been intensively managed [84]. Also the imposition of real land use patterns could only partly remove this mismatch because the land use data only distinguished forest and non-forest areas, without tree species-specific information. As a result, the simulated distribution was maintained in the simulated data as long as the land use data indicated that the forest cover was, at least, 10% (see Materials and Methods). Another explanation for the wider simulated ranges might be that the inventory might not cover all small outlier populations. (B) Comparison between the simulated LAI of the last five main tree species (Picea abies, Pinus halepensis, Quercus ilex, Quercus pubescens and Tilia cordata) and presence data from the Third Spanish Forestry Inventory (IFN = Inventario Forestal Nacional). (TIF) [file pone.0029373.s001.tif]
