# Supplementary material for: The Contribution of Vegetation and Landscape Configuration for Predicting Environmental Change Impacts on Iberian Birds
Source: PLoS One. 2011 Dec 22;6(12):e29373. doi: 10.1371/journal.pone.0029373 (PMC3245269; doi:10.1371/journal.pone.0029373)
Supplement: Table S1 — Main habitats (G = grassland, S = shrubland, F = forest, O = others) for the 168 bird species included in the study. The information was gathered from the Spanish Atlas of Breeding Birds [33] and complemented by consultation with the following experts: Carlos Ponce, Sergio Pérez Gil and Alejandro Aparicio Valenciano. (DOCX) [file pone.0029373.s002.docx]

**Table S1.**

| **Common name** | **Scientific name** | **Main habitat** |
| --- | --- | --- |
| Goshawk | *Accipiter gentilis* | F |
| Eurasian Sparrowhawk | *Accipiter nisus* | F |
| Long-tailed Tit | *Aegithalos caudatus* | F |
| Tengmalm's Owl | *Aegolius funereus* | F |
| Eurasian Black Vulture | *Aegypius monachus* | F |
| Skylark | *Alauda arvensis* | G/S |
| Common Kingfisher | *Alcedo atthis* | F |
| Red-legged Partridge | *Alectoris rufa* | G/S |
| Tawny Pipit | *Anthus campestris* | G/S |
| Water Pipit | *Anthus spinoletta* | G/S |
| Tree Pipit | *Anthus trivialis* | S/F |
| Common Swift | *Apus apus* | O |
| White-rumped Swift | *Apus caffer* | O |
| Pallid Swift | *Apus pallidus* | O |
| Iberian Imperial Eagle | *Aquila adalberti* | S/F |
| Golden Eagle | *Aquila chrysaetos* | G/S/F |
| Short-eared Owl | *Asio flammeus* | G |
| Long-eared Owl | *Asio otus* | S/F |
| Little Owl | *Athene noctua* | S/F |
| Eurasian Eagle Owl | *Bubo bubo* | S/F |
| Cattle Egret | *Bubulcus ibis* | G/S |
| Eurasian Thick-knee | *Burhinus oedicnemus* | G |
| Common Buzzard | *Buteo buteo* | G/S/F |
| Greater Short-toed Lark | *Calandrella brachydactyla* | G |
| Lesser Short-toed Lark | *Calandrella rufescens* | G/S |
| European Nightjar | *Caprimulgus europaeus* | F |
| Red-necked Nightjar | *Caprimulgus ruficollis* | S/F |
| Linnet | *Carduelis cannabina* | G/S |
| European Goldfinch | *Carduelis carduelis* | G/S |
| European Greenfinch | *Carduelis chloris* | S/F |
| Eurasian Siskin | *Carduelis spinus* | F |
| Rufous-tailed Scrub-robin | *Cercotrichas galactotes* | S |
| Short-toed Treecreeper | *Certhia brachydactyla* | F |
| Common Treecreeper | *Certhia familiaris* | F |
| Cetti's Warbler | *Cettia cetti* | O |
| Dupont's Lark | *Chersophilus duponti* | S |
| White Stork | *Ciconia ciconia* | G/F |
| Black Stork | *Ciconia nigra* | F |
| Short-toed Snake-eagle | *Circaetus gallicus* | F |
| Northern Harrier | *Circus cyaneus* | G/S |
| Montagu's Harrier | *Circus pygargus* | G/S |
| Zitting Cisticola | *Cisticola juncidis* | G |
| Great Spotted Cuckoo | *Clamator glandarius* | S |
| Hawfinch | *Coccothraustes coccothraustes* | S/F |
| Stock Dove | *Columba oenas* | G/S/F |
| Common Wood-pigeon | *Columba palumbus* | F |
| European Roller | *Coracias garrulus* | S/F |
| Common Raven | *Corvus corax* | F |
| Carrion Crow | *Corvus corone* | F |
| Jackdaw | *Corvus monedula* | G/S/F |
| Common Quail | *Coturnix coturnix* | G/S |
| Common Cuckoo | *Cuculus canorus* | F |
| Azure-winged Magpie | *Cyanopica cyana* | F |
| House Martin | *Delichon urbica* | O |
| Great Spotted Woodpecker | *Dendrocopos major* | F |
| Middle Spotted Woodpecker | *Dendrocopos medius* | F |
| Lesser Spotted Woodpecker | *Dendrocopos minor* | F |
| Black Woodpecker | *Dryocopus martius* | F |
| Black-winged Kite | *Elanus caeruleus* | G/S |
| Corn Bunting | *Emberiza calandra* | G/S |
| Rock Bunting | *Emberiza cia* | F |
| Cirl Bunting | *Emberiza cirlus* | F |
| Yellowhammer | *Emberiza citrinella* | S |
| Ortolan Bunting | *Emberiza hortulana* | S |
| European Robin | *Erithacus rubecula* | S/F |
| Lesser Kestrel | *Falco naumanni* | G |
| Peregrine Falcon | *Falco peregrinus* | O |
| Eurasian Hobby | *Falco subbuteo* | S/F |
| Common Kestrel | *Falco tinnunculus* | G/S/F |
| European Pied Flycatcher | *Ficedula hypoleuca* | F |
| Chaffinch | *Fringilla coelebs* | F |
| Crested Lark | *Galerida cristata* | G/S |
| Thekla Lark | *Galerida theklae* | S |
| Eurasian Jay | *Garrulus glandarius* | F |
| Bearded Vulture | *Gypaetus barbatus* | O |
| Griffon vulture | *Gyps fulvus* | G/S/F |
| Bonelli's Eagle | *Hieraaetus fasciatus* | G/S/F |
| Booted Eagle | *Hieraaetus pennatus* | F |
| Eastern Olivaceous Warbler | *Hippolais pallida* | S |
| Melodious Warbler | *Hippolais polyglotta* | S |
| Red-rumped Swallow | *Hirundo daurica* | G/S/F |
| Eurasian Wryneck | *Jynx torquilla* | F |
| Rock Ptarmigan | *Lagopus mutus* | G |
| Red-backed Shrike | *Lanius collurio* | S/F |
| Southern Grey Shrike | *Lanius meridionalis* | S/F |
| Woodchat Shrike | *Lanius senator* | S/F |
| Red Crossbill | *Loxia curvirostra* | F |
| Woodlark | *Lullula arborea* | G/S |
| Common Nightingale | *Luscinia megarhynchos* | S |
| Bluethroat | *Luscinia svecica* | G/S |
| Calandra Lark | *Melanocorypha calandra* | G |
| European Bee-eater | *Merops apiaster* | S |
| Black Kite | *Milvus migrans* | F |
| Red Kite | *Milvus milvus* | F |
| Rufous-tailed Rock-Thrush | *Monticola saxatilis* | O |
| Blue Rock-Thrush | *Monticola solitarius* | O |
| White-winged Snowfinch | *Montifringilla nivalis* | G |
| White Wagtail | *Motacilla alba* | G |
| Grey Wagtail | *Motacilla cinerea* | O |
| Yellow Wagtail | *Motacilla flava* | G |
| Spotted Flycatcher | *Muscicapa striata* | S/F |
| Egyptian Vulture | *Neophron percnopterus* | G/S |
| Black-eared Wheatear | *Oenanthe hispanica* | G/S |
| Black Wheatear | *Oenanthe leucura* | O |
| Wheatear | *Oenanthe oenanthe* | G/S |
| Golden Oriole | *Oriolus oriolus* | F |
| Great Bustard | *Otis tarda* | G |
| Scops Owl | *Otus scops* | S/F |
| Coal Tit | *Parus ater* | F |
| Blue Tit | *Parus caeruleus* | F |
| European Crested Tit | *Parus cristatus* | F |
| Great Tit | *Parus major* | F |
| Marsh Tit | *Parus palustris* | F |
| House Sparrow | *Passer domesticus* | G |
| Spanish Sparrow | *Passer hispaniolensis* | G |
| Tree Sparrow | *Passer montanus* | G/S |
| Grey Partridge | *Perdix perdix* | S |
| European Honey-buzzard | *Pernis apivorus* | F |
| Rock Sparrow | *Petronia petronia* | G/F |
| Black Redstart | *Phoenicurus ochruros* | S |
| Redstart | *Phoenicurus phoenicurus* | G/S |
| Western Bonelli's Warbler | *Phylloscopus bonelli* | F |
| Chiffchaff | *Phylloscopus collybita* | F |
| Magpie | *Pica pica* | S/F |
| Eurasian Green Woodpecker | *Picus viridis* | F |
| Alpine Accentor | *Prunella collaris* | S |
| Dunnock | *Prunella modularis* | G/S |
| Pin-tailed Sandgrouse | *Pterocles alchata* | G |
| Black-bellied Sandgrouse | *Pterocles orientalis* | G |
| Eurasian Crag Martin | *Ptyonoprogne rupestris* | O |
| Alpine Chough | *Pyrrhocorax graculus* | G |
| Red-billed Chough | *Pyrrhocorax pyrrhocorax* | G |
| Bullfinch | *Pyrrhula pyrrhula* | F |
| Firecrest | *Regulus ignicapilla* | F |
| Goldcrest | *Regulus regulus* | F |
| Penduline Tit | *Remiz pendulinus* | O |
| Sand Martin | *Riparia riparia* | O |
| Whinchat | *Saxicola rubetra* | G/S |
| African Stonechat | *Saxicola torquata* | G/S |
| Woodcock | *Scolopax rusticola* | F |
| Citril Finch | *Serinus citrinella* | F |
| European Serin | *Serinus serinus* | F |
| Nuthatch | *Sitta europaea* | F |
| Eurasian Collared Dove | *Streptopelia decaocto* | O |
| Turtle Dove | *Streptopelia turtur* | F |
| Tawny Owl | *Strix aluco* | G/F |
| Spotless Starling | *Sturnus unicolor* | O |
| Starling | *Sturnus vulgaris* | G |
| Black-capped Warbler | *Sylvia atricapilla* | S/F |
| Garden Warbler | *Sylvia borin* | S/F |
| Subalpine Warbler | *Sylvia cantillans* | S |
| Whitethroat | *Sylvia communis* | S |
| Spectacled Warbler | *Sylvia conspicillata* | S |
| Orphean Warbler | *Sylvia hortensis* | G/S |
| Sardinian Warbler | *Sylvia melanocephala* | S |
| Dartford Warbler | *Sylvia undata* | S |
| Alpine Swift | *Tachymarptis melba* | O |
| Capercaillie | *Tetrao urogallus* | G/S |
| Little Bustard | *Tetrax tetrax* | G |
| Wallcreeper | *Tichodroma muraria* | O |
| Winter Wren | *Troglodytes troglodytes* | S/F |
| Blackbird | *Turdus merula* | S/F |
| Song Thrush | *Turdus philomelos* | S/F |
| Ring Ouzel | *Turdus torquatus* | S |
| Mistle Thrush | *Turdus viscivorus* | F |
| [Barn Owl](http://en.wikipedia.org/wiki/Barn_Owl) | *Tyto alba* | G |
| Hoopoe | *Upupa epops* | G/S/F |
| Northern Lapwing | *Vanellus vanellus* | G |
